# ADAMTS1-mediated targeting of TSP-1 by PPARδ suppresses migration and invasion of breast cancer cells

**DOI:** 10.18632/oncotarget.21584

**Published:** 2017-10-06

**Authors:** Sun Ah Ham, Taesik Yoo, Won Jin Lee, Jung Seok Hwang, Jinwoo Hur, Kyung Shin Paek, Dae-Seog Lim, Sung Gu Han, Chi-Ho Lee, Han Geuk Seo

**Affiliations:** ^1^ Sanghuh College of Life Sciences, Konkuk University, Seoul 05029, Korea; ^2^ Department of Nursing, Semyung University, Jechon 27136, Korea; ^3^ Department of Biotechnology, CHA University, Seongnam 13488, Korea

**Keywords:** a disintegrin and metalloprotease domains with thrombospondin motifs 1, breast cancer cells, metastasis, peroxisome proliferator-activated receptor δ, thrombospondin-1

## Abstract

Migration and invasion of cancer cells into surrounding tissue is a key stage of cancer metastasis. Here, we show that peroxisome proliferator-activated receptor (PPAR) δ regulates migration and invasion of human breast cancer cells via thrombospondin-1 (TSP-1) and its degrading protease, a disintegrin and metalloprotease domains with thrombospondin motifs 1 (ADAMTS1). Activation of PPARδ by GW501516, a specific ligand for PPARδ, led to marked inhibition in the cell migration and TSP-1 expression of breast cancer. These effects were suppressed by small interfering RNA-mediated knock-down of ADAMTS1, indicating that ADAMTS1 is involved in PPARδ-mediated inhibition of migration and TSP-1 expression in breast cancer cells. In addition, ligand-activated PPARδ upregulated expression of ADAMTS1 at the transcriptional level via binding of PPARδ to a direct repeat-1 site within the ADAMTS1 gene promoter. Furthermore, ligand-activated PPARδ suppressed invasion of breast cancer cells in an ADAMTS1-dependent manner. Taken together, these results demonstrate that PPARδ suppresses migration and invasion of breast cancer cells by downregulating TSP-1 in a process mediated by upregulation of ADAMTS1.

## INTRODUCTION

Peroxisome proliferator-activated receptor δ (PPARδ), a ligand-inducible transcription factor belonging to the nuclear hormone receptor superfamily, mediates a number of processes, including energy metabolism, cellular growth and differentiation, and inflammatory responses [1]. PPARδ promotes transcription of target genes by forming heterodimers with the retinoid X receptor via PPAR response elements (PPRE), which contain a direct repeat (DR1 or DR2) comprising six nucleotides (AGGTCA) separated by one or two nucleotides, present in the promoter region of target genes [2, 3]. In addition to its normal homeostatic functions, PPARδ also plays roles in a number of disease processes, including type 2 diabetes, hyperlipidemia, atherosclerotic inflammation, and oncogenesis [1]. Although PPARδ is expressed by several tumor-derived cell lineages and primary human tumors, including liver, prostate, colon, gastric, and breast cancers, its exact role in cancer development is unclear. Several studies demonstrate that activation of PPARδ increases proliferation and tumorigenesis of melanoma, liver, intestinal adenoma, and prostate cancer cell lines [4-9]. Others, however, report that PPARδ agonists inhibit proliferation of skin cancer, colon carcinoma, and lung adenocarcinoma [10-12]. Furthermore, recent studies show that ligand-activated PPARδ inhibits proliferation of human breast and melanoma cancer cell lines [13, 14].

Thrombospondin-1 (TSP-1) is a 450 kDa oligomeric extracellular matrix glycoprotein that regulates several biological processes, including angiogenesis, cell adhesion, and tumor progression [15, 16]. Initially, TSP-1 was considered a potent inhibitor of tumor progression because increased TSP-1 expression suppresses growth or metastasis of human melanoma *in vivo* [17]. However, this effect appears to be cell type-specific, as demonstrated by a study showing that TSP-1 stimulates epithelial-to-mesenchymal transition in human melanoma and breast carcinoma cells, leading to an aggressive phenotype [18]. Although the role of TSP-1 in melanoma is controversial, it has a carcinogenic effect that promotes metastasis and progression of breast cancer [16, 19, 20].

ADAMTS1 (a disintegrin and metalloprotease with thrombospondin motifs 1) is a zinc-binding metalloprotease broadly expressed by several adult tissues, and is implicated in tissue remodeling during cancer development and progression [21-23]. In fact, upregulation of ADAMTS1 occurs in highly metastatic pancreatic cancers [24]. By contrast, other studies provide conflicting evidence of its expression in human breast tumors [25, 26]. Accordingly, ADAMTS1 is considered to be both a pro- and an anti-tumorigenic factor, although the specific mechanisms underlying these are poorly understood.

Although the role of PPARδ in the tumorigenicity of breast cancer is controversial, recent reports have shown the anti-proliferative activities of PPARδ in breast cancer cells [13, 14]. Thus, we hypothesized that ligand-activated PPARδ plays a central role in the tumorigenicity of human breast cancers, by modulating the expression of TSP-1 through its degrading protease ADAMTS1. Here, we examined the association between TSP-1 and ADAMTS1 and the activity of ligand-activated PPARδ in terms of migration and invasion of human breast cancer cells. We show that activation of PPARδ by GW501516 inhibits migration and invasion of breast cancer cells, and that PPARδ exerts its inhibitory effects by downregulating TSP-1 expression in a process mediated by transcriptional upregulation of ADAMTS1.

## RESULTS

### Activation of PPARδ suppresses migration of breast cancer cells

**Figure 1:**
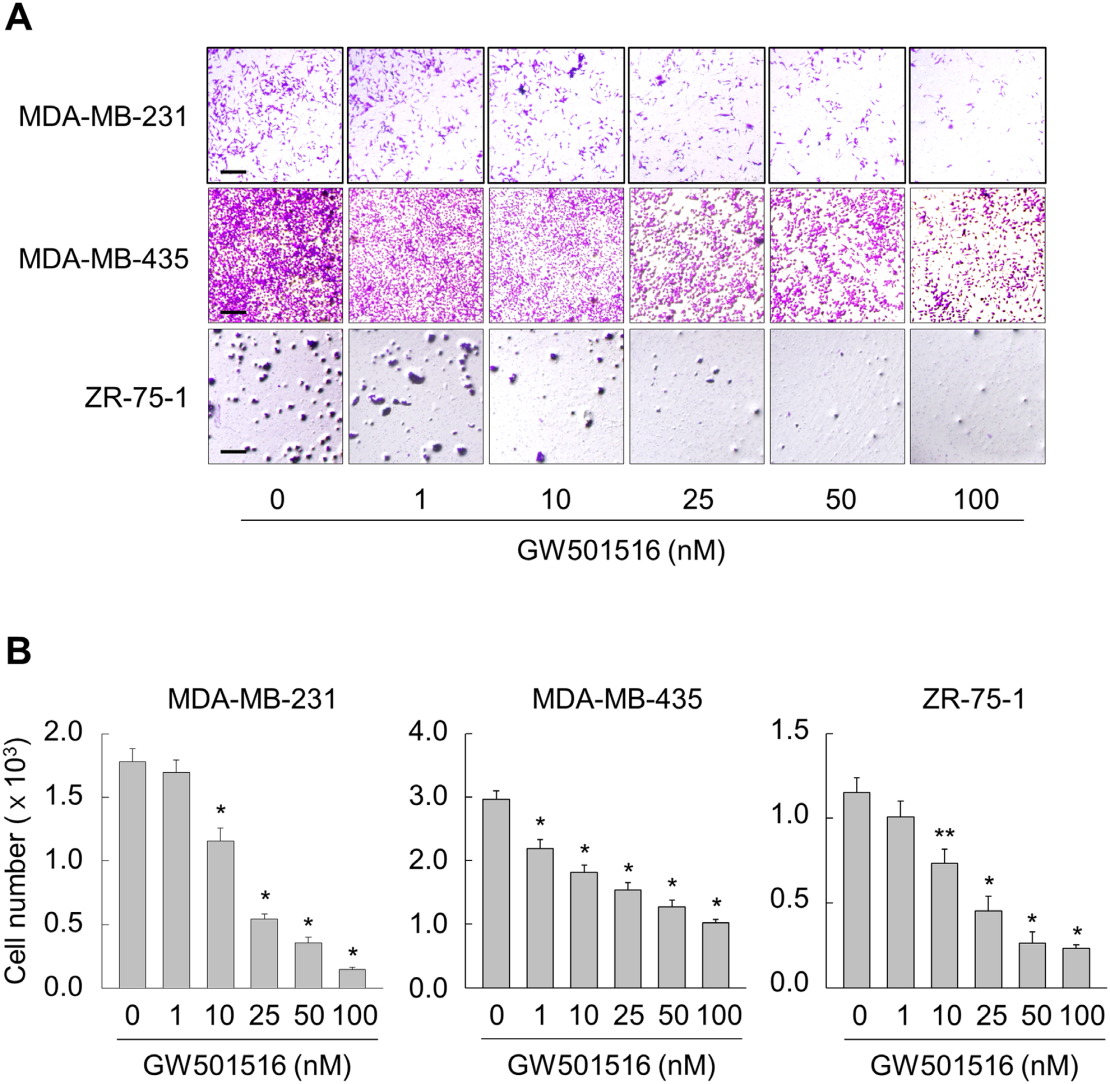
Activating PPARδ inhibits migration of MDA-MB-231, MDA-MB-435, and ZR-75-1 cells **(A, B)** Cells were incubated with various concentrations of GW501516. After 48 h, cells were examined in migration assays (A) and migrating cells were quantitated (B). Representative images from four independent experiments are shown. Results are expressed as the mean ± SE (n = 4). Bar, 100 μm. ^*^p < 0.01, ^**^p < 0.05 compared with the untreated group.

First, we examined the effects of GW501516 on migration of human breast cancer cell lines MCF-7 and MDA-MB-231, which show low and high metastatic potential, respectively. When GW501516 was added to the culture medium of MCF-7 and MDA-MB-231 cells, it inhibited migration of the latter but not the former (Supplementary Figure 1). The inhibitory activity against MDA-MB-231 was concentration-dependent and was apparent at concentrations as low as 10 nM, reaching maximal inhibition at 100 nM. Consistent with the results in MDA-MB-231 cells, the migration of other high metastatic human breast cancer cell lines MDA-MB-435 and ZR-75-1 was dose-dependently inhibited in the presence of GW501516 (Figure 1).

### Activation of PPARδ inhibits TSP-1 expression in breast cancer cells

**Figure 2:**
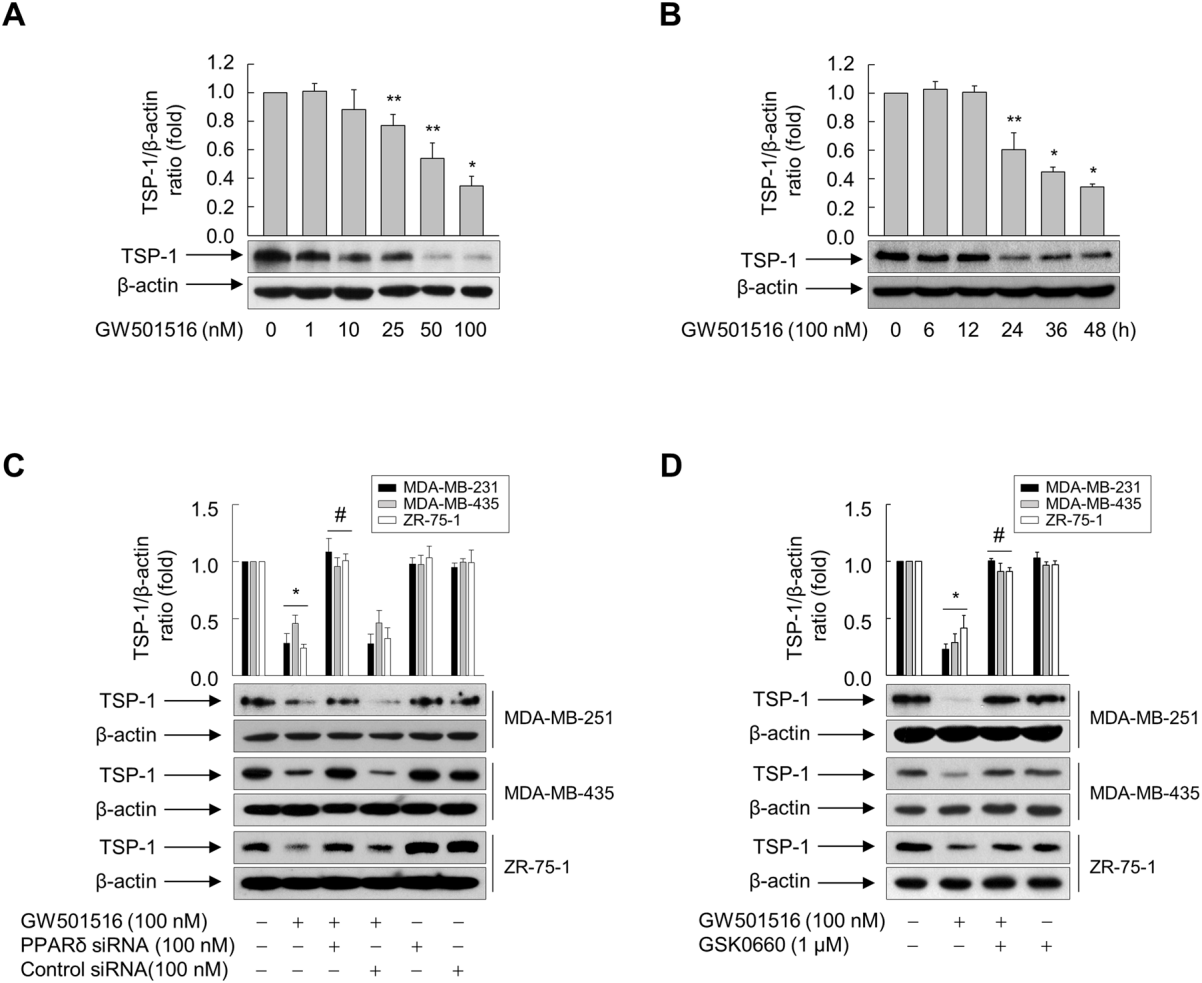
Activating PPARδ inhibits TSP-1 expression in MDA-MB-231, MDA-MB-435, and ZR-75-1 cells **(A)** MDA-MB-231 cells were incubated with various concentrations of GW501516 for 48 h. **(B)** MDA-MB-231 cells were treated with 100 nM GW501516 for the indicated times. **(C, D)** Cells transfected with siRNA specific for PPARδ or with control siRNA for 24 h (C) or pretreated with GSK0660 for 30 min (D) were exposed to GW501516 for 48 h. The cells were then harvested, and an aliquot of total cell lysate was analyzed by Western blotting. Representative blots from three independent experiments are shown. Results are expressed as the mean ± SE (n = 3). ^*^p < 0.01 and ^**^p < 0.05, compared with the untreated group; ^#^*p* < 0.01, compared with the GW501516-treated group.

To examine the mechanisms underlying GW501516-mediated inhibition of breast cancer cell migration, we targeted TSP-1, which induces tumor metastasis in human breast cancer [20]. Basal expression of TSP-1 in MDA-MB-231 cells was higher than that in MCF-7 cells (Supplementary Figure 2). Next, we exposed MDA-MB-231 cells to GW501516 to further confirm whether GW501516-mediated suppression of MDA-MB-231 cell migration was dependent on TSP-1. We found that TSP-1 expression decreased significantly and in a time- and dose-dependent manner. Maximum inhibitory activity was obtained after 48 h of exposure to 100 nM GW501516 (Figure 2A). When cells were treated with 100 nM GW501516, significant inhibition of TSP-1 protein levels was detected at 24 h, reaching a maximum at 48 h (Figure 2B).

To further elucidate the role of PPARδ in GW501516-mediated inhibition of TSP-1 expression, we transfected MDA-MB-231 cells with either small interfering (si)RNA specific for PPARδ or control siRNA. PPARδ expression fell significantly in the presence of PPARδ siRNA, but was unchanged in the presence of nonspecific control siRNA (Supplementary Figure 3). This siRNA-mediated downregulation of PPARδ counteracted GW501516-mediated inhibition of expression of TSP-1, whereas control siRNA had no effect on TSP-1 levels (Figure 2C). In addition, pretreating cells with GSK0660, an antagonist of PPARδ [27], reversed the effect of GW501516 on TSP-1 expression (Figure 2D). Similar results are also observed in MDA-MB-435 and ZR-75-1 cells (Figure 2C and 2D). These data clearly indicate that PPARδ is involved in GW501516-mediated inhibition of TSP-1 expression in breast cancer cells.

### Activation of PPARδ inhibits migration and TSP-1 expression through ADAMTS1

**Figure 3:**
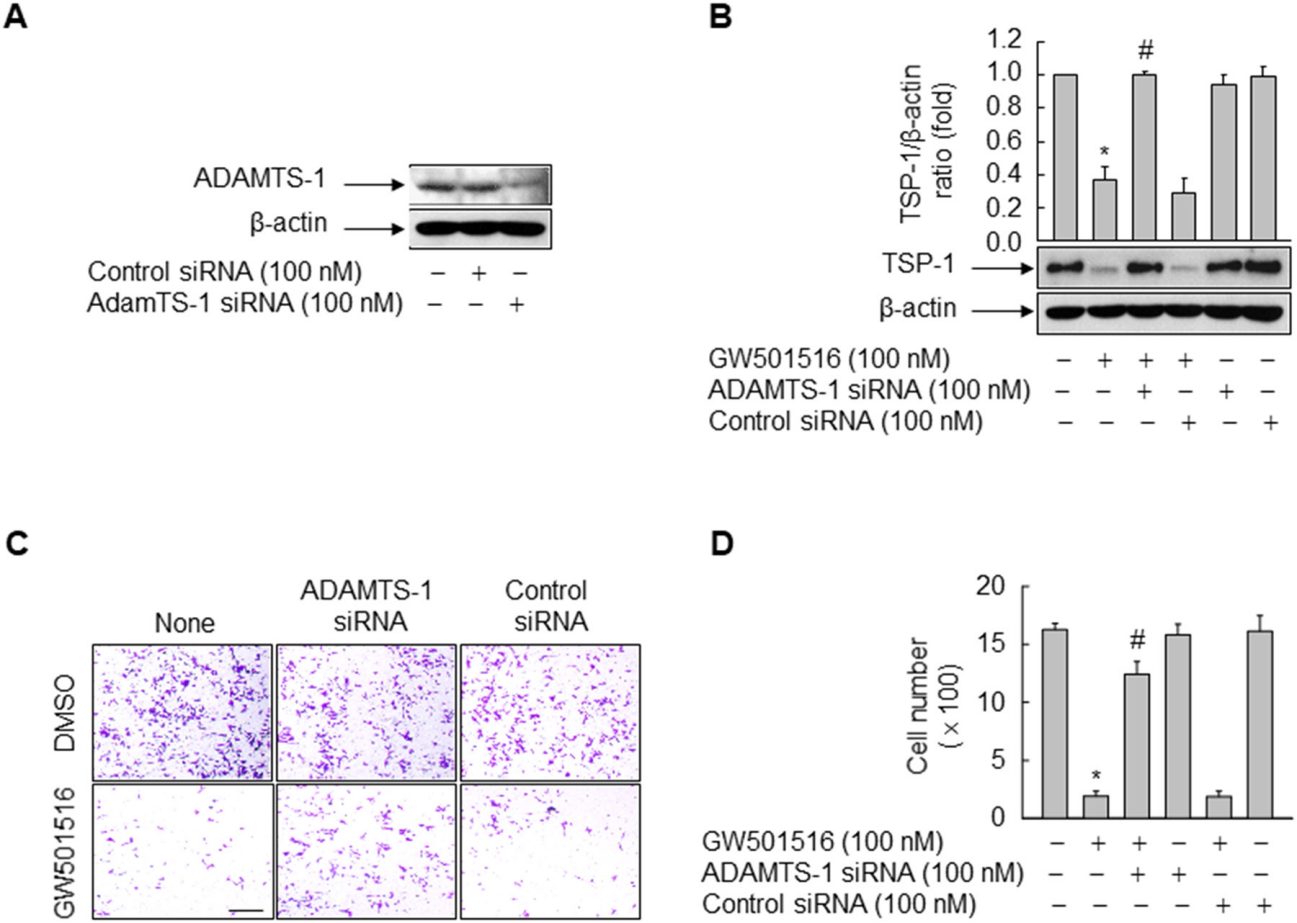
Activating PPARδ inhibits cells migration and TSP-1 expression in MDA-MB-231 cells via ADAMTS1 **(A)** Cells were transfected with siRNA specific for ADAMTS1 or with control siRNA. Following incubation for 24 h, cells were harvested and the effects of siRNA were evaluated by Western blot analysis. **(B)** Cells transfected with siRNA specific for ADAMTS1 or with control siRNA for 24 h were treated with or without 100 nM GW501516. After 48 h, cells were harvested and TSP-1 levels were analyzed by Western blotting. **(C, D)** Cells transfected with siRNA specific for ADAMTS1 or with control siRNA for 24 h were treated with 100 nM GW501516 or vehicle (DMSO). After 48 h, cells were examined in migration assays (C) and migrating cells were counted (D). Representative blots or images from three or four independent experiments are shown. Results are expressed as the mean ± SE (n = 3 or 4). Bar, 100 μm. ^*^p < 0.01, compared with the untreated group; ^#^*p* < 0.01, compared with the GW501516-treated group.

Because ADAMTS1, a zinc-binding metalloproteinase, recognizes TSP-1 as a substrate [21], we examined the role of ADAMTS1 in GW501516-induced inhibition of TSP-1 in MDA-MB-231 cells. siRNA-mediated downregulation of ADAMTS1 counteracted the inhibitory effects of GW501516 on TSP-1 expression; these effects were not observed in the presence of control siRNA (Figure 3A and 3B).

To confirm whether ADAMTS1-mediated inhibition of TSP-1 has a direct effect on GW501516-regulated cell migration, we performed a cell migration assay using the Transwell system and cells transfected with siRNA targeting ADAMTS1. Consistent with the effect of ADAMTS1 on TSP-1, ADAMTS1 siRNA (but not control siRNA) reversed the effects of GW501516 on cell migration (Figure 3C and 3D). These results suggest that the effects of GW501516 on the cell migration are regulated by ADAMTS1 via proteolytic degradation of TSP-1, a pro-metastatic factor, in breast cancer cells.

### Activation of PPARδ induces ADAMTS1 expression

**Figure 4:**
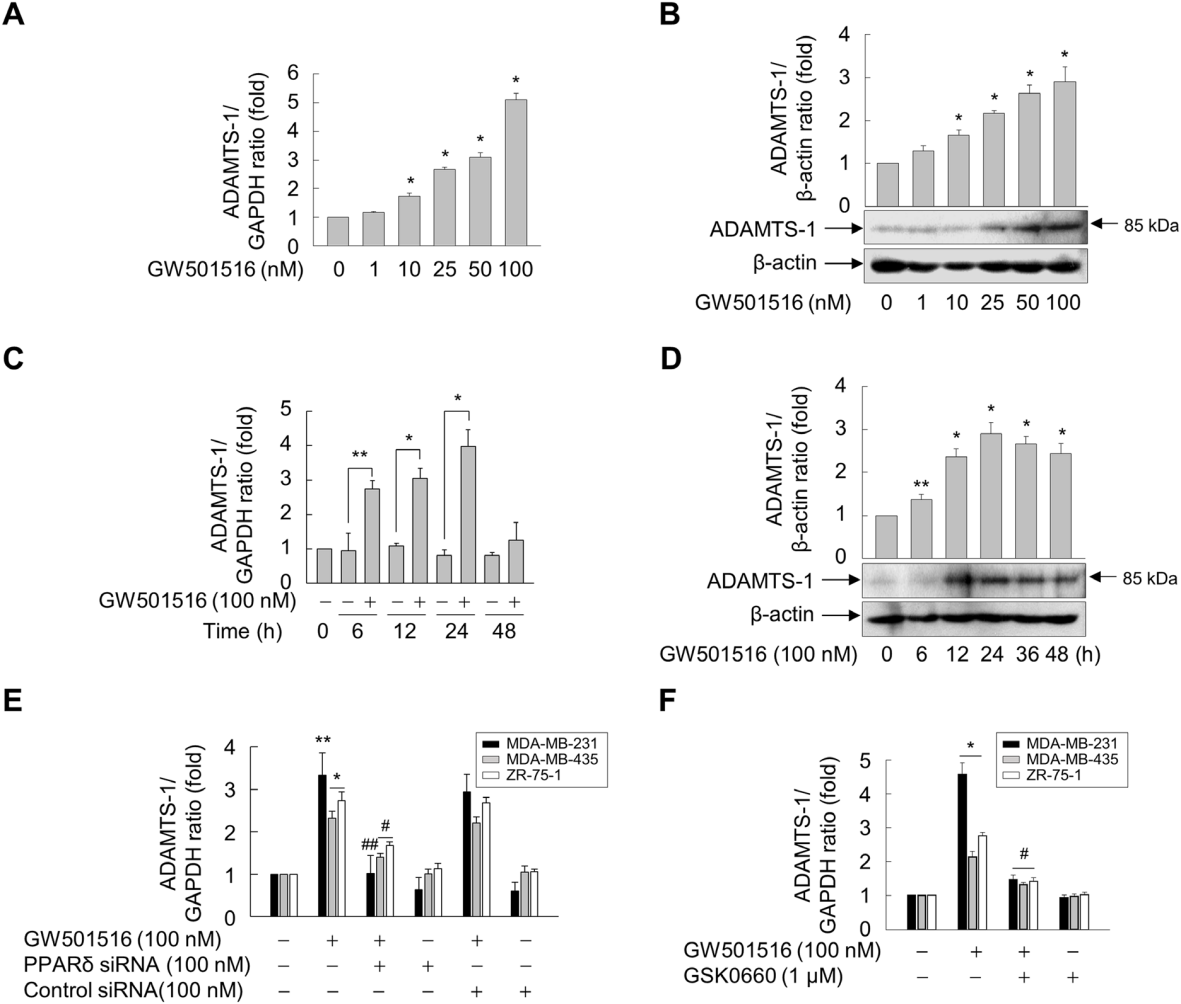
Activating PPARδ induces ADAMTS1 expression in MDA-MB-231, MDA-MB-435, and ZR-75-1 cells **(A, B)** MDA-MB-231 cells were incubated with various concentrations of GW501516 for 48 h. **(C, D)** MDA-MB-231 cells were incubated with or without 100 nM GW501516 for the indicated times. **(E, F)** Cells transfected with siRNA specific for PPARδ or with control siRNA for 24 h (E) or pretreated with GSK0660 for 30 min (F) were exposed to GW501516 for 24 h. The cells were then harvested, and levels of ADAMTS1 mRNA (A, C, E, F) and protein (B, D) were analyzed by real-time PCR and immunoblotting, respectively. Representative blots from three independent experiments are shown. Results are expressed as the mean ± SE (n = 3). ^*^p < 0.01 and ^**^p < 0.05, compared with the untreated group; ^#^*p* < 0.01 and ^##^*p* < 0.05, compared with the GW501516-treated group.

To further examine the roles of ADAMTS1 in GW501516-mediated regulation of cell migration and TSP-1 expression, we investigated the effect of PPARδ activation on expression of ADAMTS1 in MDA-MB-231 cells. When cells were treated with GW501516, the levels of ADAMTS1 mRNA and protein increased in a time- and dose-dependent manner. Maximum expression of ADAMTS1 mRNA and protein was observed after 24 h of exposure to 100 nM GW501516 (Figure 4A and 4B). When cells were treated with 100 nM GW501516, significant elevation of ADAMTS1 mRNA and protein expression was detected at 6 h, reaching a maximum at 24 h (Figure 4C and 4D).

To verify the role of PPARδ in upregulation of ADAMTS1, we examined the effect of GW501516 in cells transfected with siRNA specific for PPARδ. Downregulation of PPARδ led to marked suppression of GW501516-induced ADAMTS1 mRNA expression (Figure 4E). In addition, pretreatment with GSK0660 significantly inhibited GW501516-induced expression of ADAMTS1 mRNA (Figure 4F), confirming involvement of PPARδ in GW501516-mediated upregulation of ADAMTS1 in breast cancer cells.

### Activation of PPARδ increases the transcriptional activity of ADAMTS1

**Figure 5:**
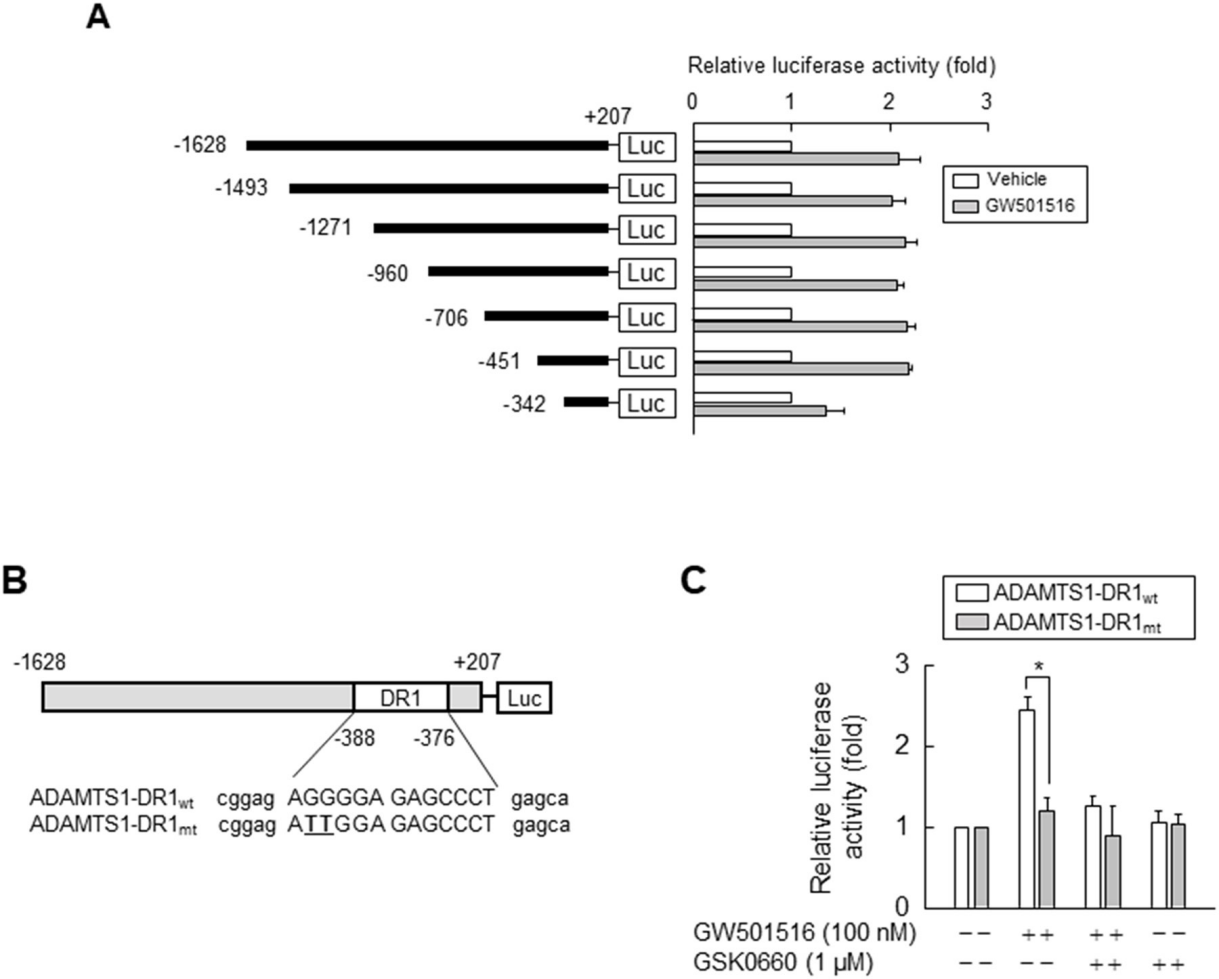
Activating PPARδ increases ADAMTS1 promoter activity via the PPRE site **(A)** HEK293T cells were transfected with ADAMTS1 gene promoter constructs (1 μg) and the SV40 β-Galactosidase expression vector (pSV β-Gal, 0.5 μg). Transfectants were grown for 24 h and then treated with 100 nM GW501516 or vehicle for 48 h. **(B)** Schematic representation of the ADAMTS1 promoter. The potential DR1-type PPRE was mutated by site-directed mutagenesis (mutated sites underlined in bold). **(C)** HEK293T cells pretreated for 30 min with GSK0660 were transfected with luciferase reporter plasmids (1 μg) driven by the ADAMTS1 promoter and with a pSV β-Gal plasmid (0.5 μg). Transfectants were grown for 24 h and then treated with GW501516 for 48 h. Luciferase activity was determined and normalized to β-galactosidase activity. Data are expressed as the mean ± SE of four independent transfections (n = 4). ^*^p < 0.01, compared with the untreated group; ^#^*p* < 0.01 compared with the GW501516-treated group.

To confirm whether GW501516-induced upregulation of ADAMTS1 occurs at the transcriptional level, we performed a reporter gene assay using a construct driven by the ADAMTS1 promoter. HEK293T cells were co-transfected with a luciferase expression vector carrying the -1.6 kb upstream region of the human ADAMTS1 gene, along with an expression vector for β-galactosidase. Following incubation for 24 h, cells were treated with vehicle or GW501516 for 48 h. As shown in Figure 5A, GW501516 increased luciferase activity, supporting the previous finding that it induces expression of ADAMTS1 mRNA and protein. We then identified the promoter region responsible for GW501516-induced upregulation of ADAMTS1 using reporter assays and serially truncated constructs driven by the ADAMTS1 promoter. The response to GW501516 was markedly reduced by deletion of sequences between nucleotides -451 and -342 (relative to the transcriptional start site at +1) of the ADAMTS1 promoter, indicating that the element responsible for the effects of GW501516 is located between -451 and -342 bases upstream of the ADAMTS1 gene.

To further confirm that this -451/-342 region of the ADAMTS1 gene is responsible for the ability of PPARδ (activated by GW501516) to induce expression of ADAMTS1, we searched a database for PPRE homolog sequences in the -451 to -342 base region. A putative PPRE containing a direct repeat-1 (DR1) sequence was identified at positions -388 to -376 within the ADAMTS1 promoter (Figure 5B). To determine whether this putative PPRE is involved in PPARδ-mediated transcriptional activation, we introduced mutations into the DR1 site of the full-length ADAMTS1 promoter (-1628/+207). Activation of PPARδ by GW501516 resulted in a 2.5-fold increase in transcriptional activity when compared with wild-type ADAMTS1-DR1 (ADAMTS1-DR1_wt_). This increase in promoter activity was significantly attenuated in the presence of GSK0660. By contrast, the effect of GW501516 on the promoter activity of ADAMTS1 was almost completely abolished by mutation of the motif within ADAMTS1-DR1 (ADAMTS1-DR1_mt_; Figure 5C). These results clearly demonstrate that the ADAMTS1 promoter is transcriptionally regulated by PPARδ through the DR1-type PPRE present upstream of the ADAMTS1 gene.

### PPARδ is recruited to the ADAMTS1 promoter upon GW501516 treatment

**Figure 6:**
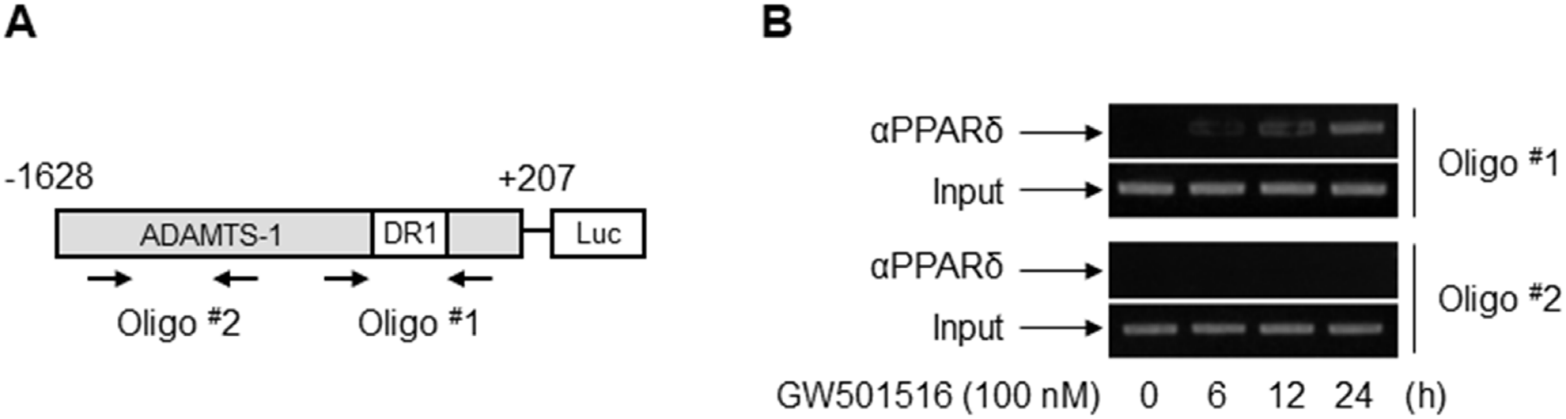
PPARδ associates physically with the PPRE within the ADAMTS1 promoter in MDA-MB-231 cells **(A)** Schematic location of the primer sets used for PCR amplification. Oligo #1 and Oligo #2 depict regions corresponding to the DR1-type PPRE site and an upstream region, respectively. **(B)** Cells were incubated with GW501516 for the indicated times. Chromatin was immunoprecipitated with an anti-PPARδ antibody, and genomic DNA was amplified by PCR using either the Oligo #1 or Oligo #2 primer set. Control amplifications were performed with input chromatin obtained before immunoprecipitation. Results are representative of three independent experiments.

Next, we performed chromatin immunoprecipitation assays to further confirm that PPARδ interacts directly with the ADAMTS1 promoter to trigger transcriptional upregulation. MDA-MB-231 cells were treated with GW501516 for 0, 6, 12, and 24 h, and chromatin fragments were subjected to immunoprecipitation with an anti-PPARδ antibody. Genomic DNA from the immunoprecipitates was amplified by PCR using primers specific for promoter sequences containing the putative ADAMTS1-DR1 site (Figure 6A). The association between PPARδ and the PCR-amplified region was detected in fragments amplified by primers targeting Oligo #1, whereas no reactivity was observed with fragments amplified with upstream primers targeting Oligo #2 (Figure 6B). These results are in line with the findings from the reporter gene assay, suggesting that GW501516-activated PPARδ regulates transcriptional activity of the ADAMTS1 gene.

### Activation of PPARδ suppresses invasion of breast cancer cells via ADAMTS1

**Figure 7:**
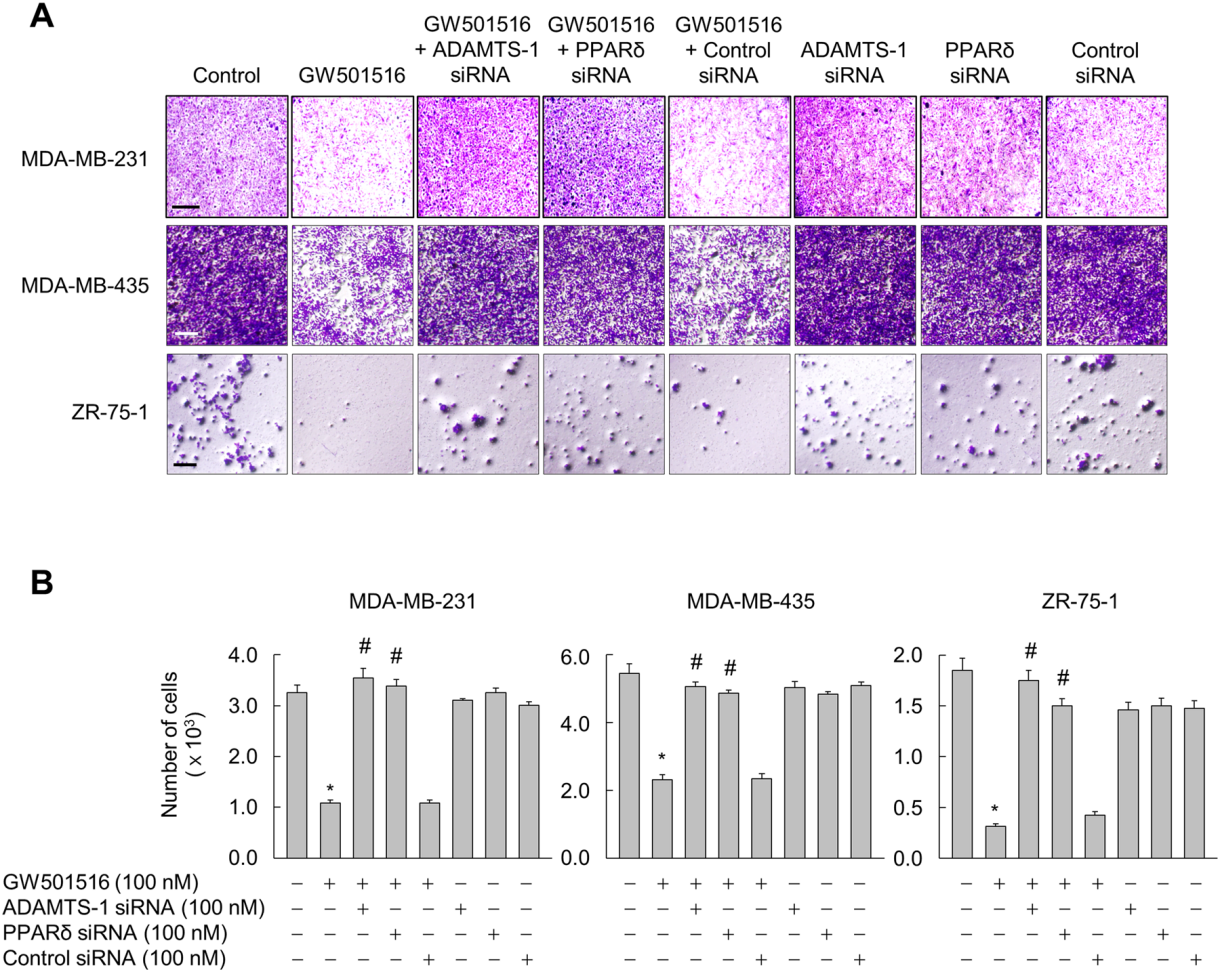
Activating PPARδ suppresses invasion of MDA-MB-231, MDA-MB-435, and ZR-75-1 cells via ADAMTS1 **(A, B)** Cells transfected with siRNA specific for ADAMTS1 or PPARδ, or with control siRNA, for 24 h were treated with 100 nM GW501516 or vehicle. Following incubation for 72 h, cells were subjected to invasion assays (A) and cells migrating to the lower chamber were counted (B). Representative images from four independent experiments are shown. Results are expressed as the mean ± SE (n = 4). Bar, 100 μm. ^*^p < 0.01, compared with the untreated group; ^#^*p* < 0.01, compared with the GW501516-treated group.

TSP-1 is reported to induce an invasive phenotype in various cancer cells [28-30], we asked whether GW501516-activated PPARδ induces phenotypic changes, such as invasion, via ADAMTS-1-mediated regulation of TSP-1 in breast cancer cells. Cells not treated with GW501516 showed a highly aggressive and invasive phenotype in a Transwell assay. This was markedly attenuated by GW501516; however, transfection of siRNA specific for ADAMTS1 or PPARδ (but not control siRNA) almost completely abolished the inhibitory effects of GW501516 (Figure 7A and 7B). These results clearly indicate that GW501516-activated PPARδ is involved in suppression of cell invasion, and that ADAMTS1 mediates the actions of PPARδ in human breast cancer cells.

## DISCUSSION

Here, we show that the GW501516-mediated activation of PPARδ suppresses migration and invasion of human breast cancers MDA-MB-231, MDA-MB-435, and ZR-75-1 cells. This effect is mediated by ADAMTS1, a metalloproteinase that utilizes the TSP-1 protein as a substrate [21]. Transfection experiments demonstrated that ADAMTS1 expression was regulated by PPARδ at the transcriptional level. Mutation of the DR1 site located within the ADAMTS1 promoter region abolished GW501516-induced ADAMTS1 transcription, indicating that this region is a novel PPRE. Furthermore, GW501516-activated PPARδ suppressed invasion of human breast cancer cells via a mechanism mediated by ADAMTS1.

Although the role of PPARδ in tumorigenesis is highly controversial, the present data demonstrated that ligand-activated PPARδ suppresses migration and invasion of human breast cancer cells. A recent study also showed that activation of PPARδ by specific ligands reduced the tumorigenicity of xenografts by manipulating expression of PPARδ [13]. In particular, activation of PPARδ negatively regulates invasion and metastasis of human pancreatic cancer cells by downregulating genes associated with these biological processes [31], thereby supporting the findings presented herein. In contrast to the present study, other studies show that administration of a PPARδ agonist, GW501516, promotes mammary tumorigenesis via changes in the inflammatory and metabolic phenotype linked to mammalian target of rapamycin (mTOR) activation or 3-phosphoinositide-dependent kinase-1 (PDK1) and Akt signaling [8, 9, 32]. Thus, the exact role of PPARδ agonists in cancer development appears to depend on the properties and doses of the ligands tested [14]. In fact, different studies report that PPARδ can promote or inhibit tumorigenesis in MCF-7 human breast cancer cells [7, 14]. However, the present data clearly demonstrate that GW501516-activated PPARδ suppresses migration and invasion of human breast cancers MDA-MB-231, MDA-MB-435, and ZR-75-1 cells.

GW501516-activated PPARδ significantly downregulates TSP-1 expression by MDA-MB-231, MDA-MB-435, and ZR-75-1 human breast cancer cells. TSP-1 promotes an aggressive phenotype in human melanoma via epithelial-to-mesenchymal transition [18], whereas it suppresses metastasis in human melanoma xenografts [17]. Although the role of TSP-1 in migration and invasion of cancer cells is unclear, other studies consistently demonstrate that TSP-1 promotes metastasis and progression of breast cancer [16, 19, 20, 33]. In particular, TSP-1 upregulates MMP-9 expression by gastric tumors, resulting in acquisition of an aggressive phenotype [34]. Consistent with our previous study demonstrating involvement of PPARδ in negative regulation of melanoma cell migration and invasion [4], we found here that PPARδ-mediated downregulation of TSP-1 is likely responsible for inhibition of aggressive breast cancer. In line with this, another study shows that ligand-activated PPARγ, another sub-member of the PPAR family, suppresses hypoxia-induced expression of TSP-1 in human pulmonary artery smooth muscle cells [35]. Accordingly, it is possible that PPARδ may also inhibit TSP-1 in other types of cancer, particularly melanoma; further studies are needed to clarify the exact role of PPARδ in expression of TSP-1.

PPARδ-induced upregulation of ADAMTS1 is a key event during PPARδ-dependent suppression in the migration and invasion of human breast cancers MDA-MB-231, MDA-MB-435, and ZR-75-1 cells. The role of ADAMTS1 in tumorigenesis is highly ambiguous due to opposing results observed by different studies [23, 26, 36, 37]. Lu et al. report that ADAMTS1 is overexpressed in breast tumors, and that this overexpression is associated with an increased risk of bone metastasis [36]. In addition, breast cancer cells induce stromal fibroblasts to secrete ADAMTS1, thereby facilitating cancer cell invasion [37]. However, ADAMTS1 suppresses migration and invasion of liver and human breast tumors [26, 38]. In particular, ADAMTS1 expression correlates positively with migration and invasion of human breast cancer MDA-MB-231 cells [26]. In line with these reports, the present study demonstrates that activation of PPARδ by a specific agonist induces expression of ADAMTS1 mRNA and protein, thereby inhibiting migration and invasion of human breast cancer cells as reported previously [26]. ADAMTS1 was originally shown to be induced during the early phase of acute myocardial infarction [39]. In hepatoma cells, expression of ADAMTS1 is regulated differentially by SP1 (specificity protein 1) and USF1 (upstream stimulatory factor 1) under normoxic and hypoxic conditions [40]. Here, we identified a novel DR1-type PPRE as a *cis*-element within the promoter region of the ADAMTS1 gene and demonstrated that it is responsible for PPARδ-mediated upregulation.

The present findings show that activation of PPARδ regulates migration and invasion of human breast cancers MDA-MB-231, MDA-MB-435, and ZR-75-1 cells by upregulating ADAMTS1 expression, thereby modulating the level of TSP-1. The effect of a PPARδ ligand (GW501516) on ADAMTS1 expression mediates the function (e.g., anti-tumor activity) of PPARδ. Accordingly, the current findings support the assumption that PPARδ is an important target for therapeutic strategies for neoplastic disorders.

## MATERIALS AND METHODS

### Materials

GW501516 and GSK0660 were purchased from Enzo Life Sciences (Farmingdale, NY, USA) and Tocris Bioscience (Bristol, UK), respectively. A monoclonal antibody specific for TSP-1 and polyclonal antibodies specific for PPARδ and ADAMTS1 were purchased from Santa Cruz Biotechnology (Dallas, TX, USA). Polyclonal rabbit anti-β-actin antibody, mitomycin C, and crystal violet were obtained from Sigma-Aldrich (St. Louis, MO, USA). Small interfering (si)RNA specific for human ADAMTS1 was purchased from Bioneer (Daejeon, Korea). Control siRNA and human PPAR siRNA were obtained from Ambion (Austin, TX, USA).

### Cell culture

Human breast cancer cell lines MCF-7, MDA-MB-231, and ZR-75-1 cells were obtained from the Korean Cell Line Bank and maintained in Roswell Park Memorial Institute medium (RPMI-1640) containing antibiotics and 10% fetal bovine serum. Human breast cancer cell line MDA-MB-435 cells (a kind gift from Dr. Hyejung Mok; Department of Bioscience and Biotechnology, Konkuk University, Seoul, Korea) were cultured in Dulbecco’s modified Eagle’s medium containing antibiotics and 10% fetal bovine serum. Human embryonic kidneyHEK293T cells were also acquired from the Korean Cell Line Bank and maintained in Dulbecco’s modified Eagle’s medium containing antibiotics and 10% fetal bovine serum. All cells were incubated in each medium at 37°C under a humidified atmosphere of 95% air and 5% CO_2_.

### Gene silencing with siRNA

Breast cancer cells were seeded into 60 mm culture dishes 18–24 h prior to transfection. Cells in serum-free medium were transfected with siRNA targeting human PPARδ, human ADAMTS1, or nonspecific sequences using Welfect-Q (WelGENE, Daegu, Korea). After incubation for 6 h, the transfection medium was replaced with fresh medium containing 10% FBS and antibiotics. After incubation for 48 h, cells were treated with the indicated reagents for the indicated times and the effects of gene silencing were assessed.

### Real-time PCR

Expression of mRNA was determined by real-time PCR as described previously [4]. Briefly, total RNA was extracted from cells using TRIzol reagent (Invitrogen, Carlsbad, CA, USA) and reverse transcribed into cDNA using the TOPscript RT DryMIX kit (Enzynomics, Seoul, Korea). Equal amounts of cDNA in a 10 μl reaction volume containing 2 × Real-Time PCR Smart mix (Solgent, Daejeon, Korea) and primers (10 pM) were amplified by real-time PCR using a MJ Research PTC-200 Thermal Cycler (Bio-Rad, Hercules, CA, USA). After an initial denaturation step for 5 min at 95°C, the reaction conditions were as follows: 40 cycles of 10 s at 95°C, 10 s at 56°C, and 10 s at 72°C. mRNA expression was normalized against that of *GAPDH* mRNA in the same sample. The following primers were used: ADAMTS1 forward, 5′-TGT GGT GTT TGC GGG GGA AAT G-3′ and reverse, 5′-TCG ATG TTG GTG GCT CCA GTT-3′; and *GAPDH* forward, 5′-CAT GGC CTT CCG TGT TCC TA-3′, and reverse, 5′-CCT GCT TCA CCA CCT TCT TGA T-3′.

### Western blot analysis

Protein expression was assessed by Western blot analysis as described previously [4]. Briefly, cells treated with the indicated reagents were washed in ice-cold phosphate-buffered saline (PBS) and lysed in PRO-PREP Protein Extraction Solution (iNtRON Biotechnology, Seoul, Korea). Aliquots of cell lysates were subjected to SDS-polyacrylamide gel electrophoresis and transferred onto a Hybond-P^+^ polyvinylidene difluoride membrane (GE Healthcare, Little Chalfont, UK). Membranes were blocked overnight at 4°C with 5% nonfat milk in Tris-buffered saline (TBS) containing 0.1% Tween-20. After a brief wash with TBS, the membranes were incubated overnight at 4°C with the indicated primary antibodies in TBS containing 0.05% Tween-20, followed by peroxidase-conjugated mouse, goat, or rabbit antibodies for 1 h at room temperature. After extensive washing in TBS containing 0.1% Tween-20, immunoreactive bands were detected using West-ZOL Plus (iNtRON Biotechnology).

### Plasmid construction and site-directed mutagenesis

Luciferase reporter constructs (pGL3-ADAMTS1) containing the promoter region of the human ADAMTS1 gene (nucleotides –1628/+207, –1493/+207, –1271/+207, –960/+207, –706/+207, –451/+207, or –342/+207) were generously provided by Dr. Satoshi Hirohata (Okayama University, Okayama, Japan). Nucleotide substitutions were introduced into the DR1 site of the ADAMTS1 promoter using the QuikChange site-directed mutagenesis kit (Stratagene, La Jolla, CA, USA). PCR amplification of the wild-type human pGL3-ADAMTS1 luciferase reporter plasmid (nucleotides –1628/+207) was performed using site-directed mutation primers (ADAMTS1-DR1_mt_: 5′-CGC TAC CGG ACG GAG ATT GGA GAG CCC TGA GCA GAG-3′ and 5′-CTC TGC TCA GGG CTC TCC AAT CTC CGT CCGGTA GCG-3′). The substituted bases are indicated in bold. PCR amplification was performed using 5 ng of template DNA; reaction conditions comprised 12 cycles of 95°C for 1 min, 55°C for 1 min, and 68°C for 7 min. PCR products were digested with *Dpn*I for 1 h at 37°C prior to transformation into competent *Escherichia coli* DH5α cells. The integrity of the constructs was verified by sequencing.

### Reporter gene assay

Cells were seeded into 6-well tissue culture plates 18–24 h prior to transfection and then co-transfected with the indicated luciferase reporter plasmids and pSV β-Gal (SV40 β-galactosidase expression vector, Promega, Madison, WI, USA) using the SuperFect reagent (Qiagen, Valencia, CA, USA). Following incubation for 24 h, cells were treated with vehicle (DMSO) or GW501516 for 48 h. Cells were then lysed in luciferase reporter lysis buffer (Promega), and luciferase activity was determined by normalizing transfection efficiency using β-galactosidase activity, as described previously [41].

### Chromatin immunoprecipitation assay

Binding of nuclear proteins to DNA was determined as described previously [41]. Briefly, nuclear proteins were cross-linked to genomic DNA by incubating them with 1% formaldehyde for 10 min at room temperature. Cells were collected (by scraping) in ice-cold PBS containing protease inhibitors and then centrifuged to obtain pellets that were resuspended in lysis buffer (1% SDS, 10 mM EDTA, and 50 mM Tris-Cl, pH 8.1) containing protease inhibitors. These were incubated on ice for 30 min. After sonication and centrifugation, the supernatants were rotated with protein G-Sepharose™ 4 Fast Flow beads for 1 h at 4°C. Following immunoprecipitation with an anti-PPARδ antibody for 72 h at 4°C, protein G-Sepharose beads were added and rotated for 2 h at 4°C. The resulting immune complexes were precipitated by centrifugation and then sequentially washed in low-salt wash buffer (0.1% SDS, 1% TritonX-100, 2 mM EDTA, 20 mM Tris-Cl, and 150 mM NaCl, pH 8.1), high-salt wash buffer (0.1% SDS, 1% TritonX-100, 2 mM EDTA, 20 mM Tris-Cl, and 500 mM NaCl, pH 8.1), LiCl wash buffer (0.25 M LiCl, 1% IGEPAL-CA630, 1% deoxycholic acid, 1 mM EDTA, and 10 mM Tris-Cl, pH 8.1), and TE buffer (10 mM Tris-Cl, 1 mM EDTA, pH 8.0). After centrifugation, the immune complexes were resuspended in 200 μl of elution buffer (1% SDS and 0.1 M NaHCO_3_) and incubated for 30 min at room temperature. After another centrifugation, the supernatant was collected and cross-linking was reversed by adding 5 M NaCl to a final concentration of 125 mM for 5 h at 65°C. The remaining proteins were digested with proteinase K, and genomic DNA fragments were recovered by phenol-chloroform extraction, followed by ethanol precipitation and resuspension in sterile H_2_O. Human genomic sequences containing the putative PPARδ-binding site (DR1, Oligo #1, −459/−157) and a region upstream of the PPRE (Oligo #2, −1237/−932) in the human ADAMTS1 promoter were amplified using the following primers: Oligo #1, 5′-TTC TTG CAC TCG CTG GAA AG-3′ and 5′-CTC CCG GAG TCA CTA AAA GG-3′; and Oligo #2, 5′-CCA GAT CAC CCA TTC CAG AA-3′ and 5′-TGA GCT AGG GCT ACA CTT TC-3′.

### Cell invasion and migration assay

Transwells were fitted with polycarbonate membranes (8 μm pores) (BD Biosciences, Franklin Lakes, NJ, USA), which were coated with collagen type I (1 mg/ml; BD Biosciences) for 1 h at 37°C. After washing once with culture medium, cells were seeded into the upper compartment of the Transwell insert (8 × 10^4^ cells/well) in culture medium containing 8 μg/ml mitomycin C (Sigma-Aldrich) to prevent proliferation. The cells were incubated for 2 h and washed with PBS. Medium containing vehicle (DMSO) or GW501516 was then added to the lower well as a chemo-attractant. After incubation for the indicated times, cells were fixed with 4% paraformaldehyde for 10 min and permeabilized with MeOH for 20 min at room temperature. After staining with 0.05% crystal violet solution for 15 min at room temperature, the upper insert of the Transwell was removed. Invading cells in the lower chamber of the Transwell insert were counted under a microscope. For the cell migration assay, cells were treated using the same procedure, except that the Transwell membrane was not coated with type I collagen.

### Statistical analysis

Data are expressed as the mean ± SE. Statistical significance was determined using Student’s *t*-test or ANOVA with post hoc (Bonferroni) correction for multiple comparisons. A value of *p* < 0.05 was considered statistically significant.

## SUPPLEMENTARY MATERIALS FIGURES



## Abbreviations

ADAMTS1: a disintegrin and metalloprotease with thrombospondin 1
DR1: direct repeat-1
mTOR: mammalian target of rapamycin
PDK1: 3-phosphoinositide-dependent kinase-1
PPARδ: peroxisome proliferator-activated receptor δ
PPRE: PPAR response elements
SP1: specificity protein 1
TSP-1: thrombospondin-1
USF1: upstream stimulatory factor 1
