# Supplementary material for: ADAMTS1-mediated targeting of TSP-1 by PPARδ suppresses migration and invasion of breast cancer cells
Source: Oncotarget. 2017 Oct 6;8(55):94091–103. doi: 10.18632/oncotarget.21584 (PMC5706858; doi:10.18632/oncotarget.21584)
Supplement: Supplementary file 1 [file oncotarget-08-94091-s001.pdf]

ADAMTS1-mediated targeting of TSP-1 by PPARδ suppresses migration and invasion of breast cancer cells

SUPPLEMENTARY MATERIALS

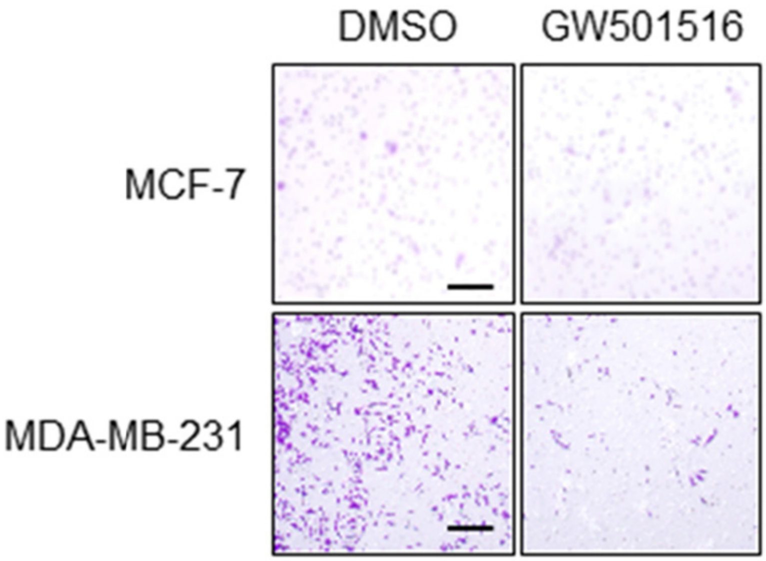

Supplementary Figure 1: Effects of GW501516 on migration of MCF-7 and MDA-MB-231 cells. Cells were treated with 100 nM GW501516 or vehicle for 48 h and then subjected to migration assays. GW501516 inhibited migration of MDA-MB-231, but not MCF-7, cells.

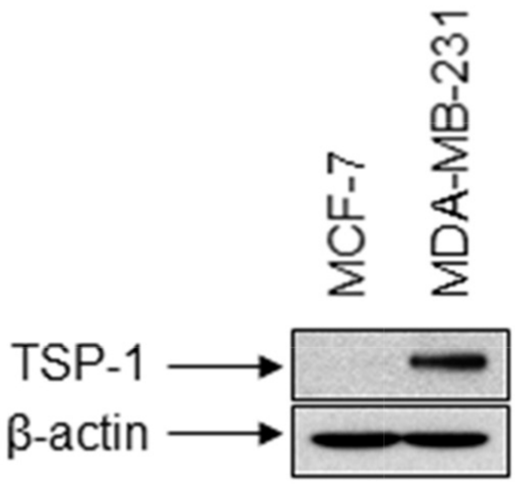

Supplementary Figure 2: Expression of TSP-1 in MCF-7 and MDA-MB-231 cells. Cells were harvested, and expression of TSP-1 protein was analyzed by Western blotting with an anti-TSP-1 antibody. MDA-MB-231 cells expressed higher levels of TSP-1 than MCF-7 cells.

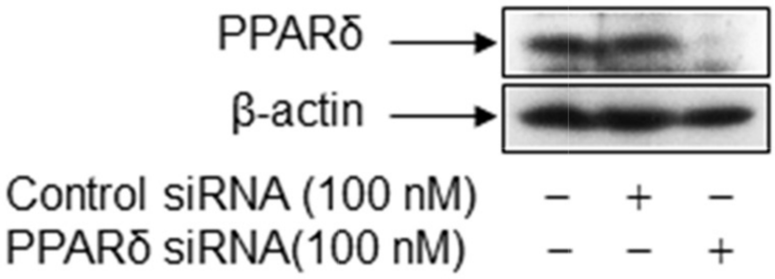

Supplementary Figure 3: Effects of siRNA on expression of PPARδ in MDA-MB-231 cells. Cells were transfected with siRNA specific for PPARδ or with control siRNA. Following incubation for 24 h, cells were harvested and an aliquot of total cell lysate was subjected to Western blot analysis. Expression of PPARδ was inhibited markedly in the presence of PPARδ siRNA, but not control siRNA.
